# Lifestyle factors, serum parameters, metabolic comorbidities, and the risk of kidney stones: a Mendelian randomization study

**DOI:** 10.3389/fendo.2023.1240171

**Published:** 2023-09-22

**Authors:** Minghui Liu, Jian Wu, Meng Gao, Yongchao Li, Weiping Xia, Youjie Zhang, Jinbo Chen, Zhiyong Chen, Zewu Zhu, Hequn Chen

**Affiliations:** ^1^ Department of Urology, Xiangya Hospital, Central South University, Changsha, China; ^2^ National Clinical Research Center for Geriatric Disorders, Xiangya Hospital, Central South University, Changsha, China; ^3^ Department of Internal Medicine, Section Endocrinology, Yale University School of Medicine, New Haven, CT, United States

**Keywords:** kidney stones, Mendelian randomization, lifestyle factors, metabolic comorbidities, mediation Mendelian randomization

## Abstract

**Background and objective:**

The early identification of modifiable risk factors is important for preventing kidney stones but determining causal associations can be difficult with epidemiological data. We aimed to genetically assess the causality between modifiable factors (lifestyle factors, serum parameters, and metabolic comorbidities) and the risk of kidney stones. Additionally, we aimed to explore the causal impact of education on kidney stones and its potential mediating pathways.

**Methods:**

We conducted a two-sample Mendelian randomization (MR) study to explore the causal association between 44 modifiable risk factors and kidney stones. The FinnGen dataset initially explored the causal relationship of risk factors with kidney stones and the UK Biobank dataset was used as the validation set. Then, a meta-analysis was conducted by combining discovery and validation datasets. We used two-step MR to assess potential mediators and their mediation proportions between education and kidney stones.

**Results:**

The combined results indicated that previous exposures may increase the risk of kidney stones, including sedentary behavior, urinary sodium, the urinary sodium/potassium ratio, the urinary sodium/creatinine ratio, serum calcium, 25-hydroxyvitamin D (25OHD), the estimated creatinine-based glomerular filtration rate (eGFRcrea), GFR estimated by serum cystatin C (eGFRcys), body mass index (BMI), waist circumference, type 2 diabetes mellitus (T2DM), fasting insulin, glycated hemoglobin, and hypertension. Coffee intake, plasma caffeine levels, educational attainment, and the urinary potassium/creatinine ratio may decrease the risk of kidney stones. Ranked by mediation proportion, the effect of education on the risk of kidney stones was mediated by five modifiable risk factors, including sedentary behavior (mediation proportion, 25.7%), smoking initiation (10.2%), BMI (8.2%), T2DM (5.8%), and waist circumference (3.2%).

**Conclusion:**

This study provides MR evidence supporting causal associations of many modifiable risk factors with kidney stones. Sedentary lifestyles, obesity, smoking, and T2DM are mediating factors in the causal relationship between educational attainment and kidney stones. Our results suggest more attention should be paid to these modifiable factors to prevent kidney stones.

## Introduction

Kidney stones is a common disease, with an overall prevalence of 1.7–14.8%, and appears to be increasing in nearly all countries (1). The annual incidence of new cases is estimated to be 15–20 per 10,000 people, 25% of whom need hospitalization (2, 3). The annual cost is predicted to double from 2 billion (2000) by 2030 in the USA (4). Unfortunately, the recurrence rate of kidney stones is up to 50% within 10 years (5). Kidney stones have been associated with a 10-year risk of a future atherosclerotic cardiovascular disease event (6). Recent population studies have found symptomatic kidney stone formers to be at increased risk for chronic and end-stage kidney disease (7, 8). Therefore, early identification and treatment of risk factors for kidney stones can help reduce the medical and financial burden.

Several lifestyle factors have been reported to be associated with kidney stones, such as smoking (9, 10), drinking (11), coffee consumption (12), sleep duration (13) and physical activity (14). Previous studies suggested that some serum and urine parameters are predictors of kidney stones, including the urinary sodium/potassium ratio (15), serum calcium (16), 25-hydroxyvitamin D (25OHD) (16), C-reactive protein (CRP) (17), urate (18), testosterone (19), estradiol (20), and lipids (21). In addition, metabolic comorbidities are highly associated with kidney stones, including obesity (22), hypertension (23), dyslipidemia (24), and diabetes (23). However, it remains difficult to measure the causal relationship between these factors and the formation of urinary stones because of limited studies, potential residual confounders, and reverse causalities. Therefore, it is of great important to disentangle whether these modifiable factors are the causations of the development of kidney stones.

Mendelian randomization (MR) is an emerging method that can be used to explore the causal relationship in the presence of potential confounders and reverse causations as it uses genetic variants as instrumental variables (IVs) and genetic variants are randomly assigned at conception (25). However, only a few risk factors of MR studies have been established to be associated with kidney stones. Here, we conducted a two-sample MR study to explore the causal relationship between kidney stones and 44 modifiable risk factors categorized as lifestyle factors, serum and urine parameters, and metabolic comorbidities. Furthermore, we conducted a meta-analysis based on the only extensive genome-wide association studies (GWASs) associated with kidney stones. Additionally, we aimed to explore the causal impact of education on kidney stones and its potential mediating pathways.

## Methods

### MR design

**Figure 1:**
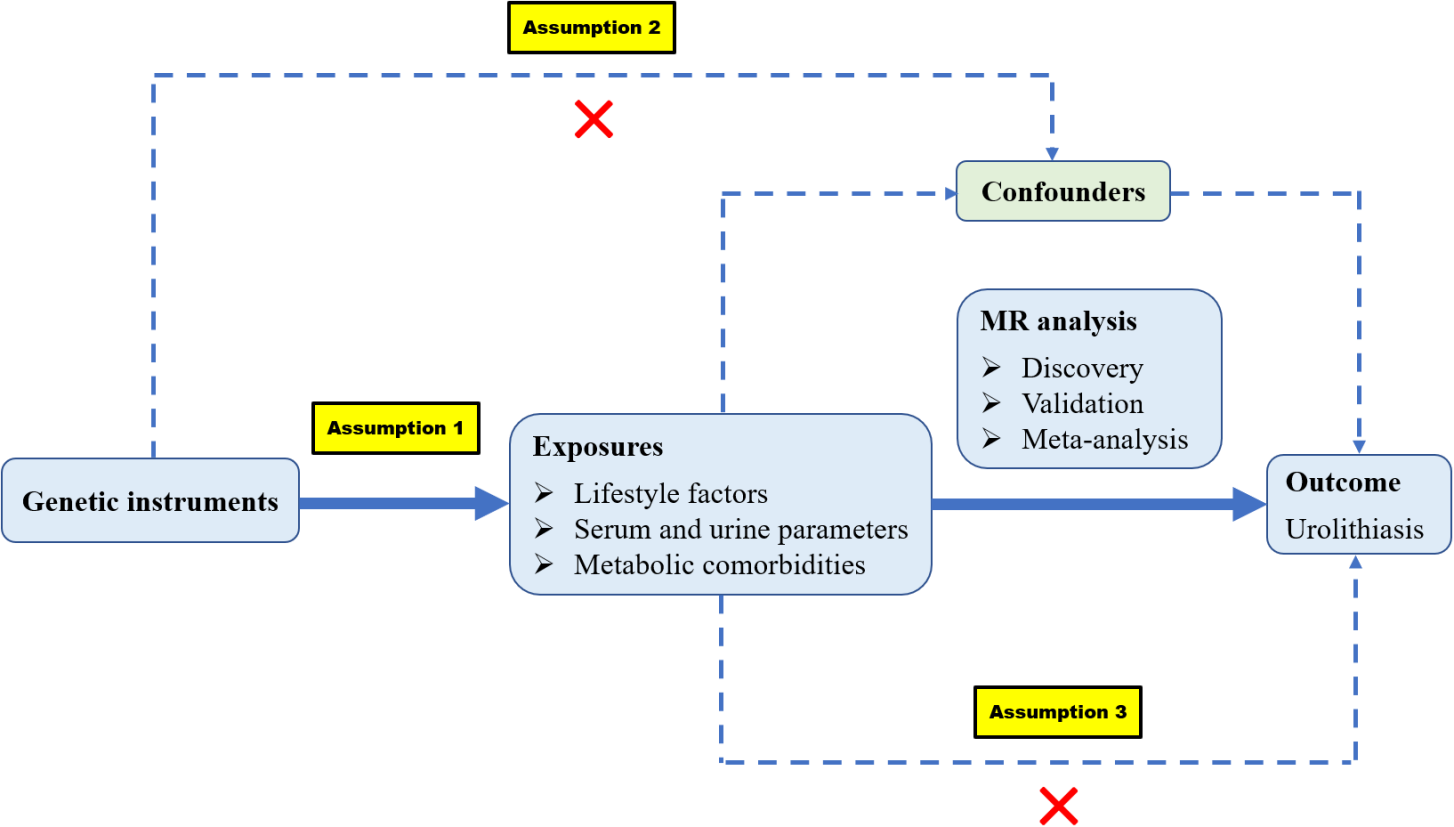
Overview of the basic assumptions and main design in our MR study.

The following are the three assumptions of MR: (1) genetic variants are robustly associated with risk factors; (2) genetic variants are not associated with any confounders; and (3) genetic variants should affect the outcome merely through the risk factors (**Figure 1**). We identified 44 modifiable risk factors that may be associated with kidney stones. These risk factors can be categorized into three groups: (1) lifestyle factors, including diet, sleep habits, physical activity, and education levels; (2) serum and urine parameters, including serum micronutrients, biochemical indices, inflammatory indices, lipid traits, sex hormones, and urinary ion excretion; and (3) metabolic comorbidities, including obesity traits, type 2 diabetes mellitus (T2DM) and related traits, hypertension and related traits, and cardiovascular diseases. The FinnGen dataset was initially used to explore the causal relationship of risk factors with kidney stones and the UK Biobank (UKBB) dataset was used as the validation set. Then, a meta-analysis was conducted by combining discovery and validation datasets.

### Selection of genetic variants

IVs were used to explore the association between modifiable risk factors and kidney stones. IVs of drinking and smoking were extracted from the GWAS and Sequencing Consortium of Alcohol and Nicotine use (GSCAN) (26). We identified the IVs of relative intakes of carbohydrate, fat, and protein from the Social Science Genetic Association Consortium (SSGAC) (27). We obtained IVs of moderate-vigorous physical activity (28), watching TV duration (sedentary behavior) (29), serum calcium (30), serum phosphate, urinary sodium (31), urinary potassium (31), 25OHD (32), the urinary sodium/potassium ratio (33), the urinary sodium/creatinine ratio (33), the urinary potassium/creatinine ratio (33), testosterone (34), and estradiol (34) based on a published GWAS from the UKBB. We extracted IVs of coffee intake (35), plasma caffeine levels (36), insomnia (37), sleep duration (37), educational attainment (38), vitamin C (39), parathyroid hormone (PTH) (40), CRP (41), the estimated creatinine-based glomerular filtration rate (eGFRcrea) (42), the GFR estimated by serum cystatin C (eGFRcys) (42), blood urea nitrogen (BUN) (42), urate (43), body mass index (BMI) (44), waist circumference (45), T2DM (46), diastolic blood pressure (DBP) (47), systolic blood pressure (SBP) (47), and coronary artery disease (CAD) (48) from some large GWAS meta-analyses. IVs of HDL cholesterol, LDL cholesterol, total cholesterol, and triglycerides were extracted from the Global Lipids Genetics Consortium (GLGC) with 188,577 European participants (49). IVs of fasting glucose and fasting insulin were obtained from Meta-Analyses of Glucose and Insulin-Related Traits (MAGIC). IVs of hypertension and ischemic stroke were extracted from the MRC Integrative Epidemiology Unit (MRC-IEU) and MEGASTROKE consortium, respectively. We extracted single nucleotide polymorphisms (SNPs) associated with each risk factor at a genome-wide significance level (P<5×10^−8^) and removed the existing linkage disequilibrium (r^2^ <0.01 and clump distance > 5,000 kb).

### GWAS summary statistics of kidney stones

We used the kidney stones GWAS summary statistics from the UKBB and FinnGen consortium (https://r7.finngen.fi/). In the UKBB, the GWAS dataset contained 6,536 cases and 388,508 controls, with adjustments for age, sex, and genotyping platform (50). We used the seventh release data from the FinnGen consortium, with 7,433 cases and 301,094 controls, with adjustments for age, sex, 10 principal components, and a genotyping batch.

### Mediation MR analysis

A two-stage MR analysis was undertaken to evaluate the mediating impact of intermediate risk factors on the causal relationships between education and the occurrence of kidney stones. First, we calculated the causal influence of genetically determined education on the mediators (β1). Second, we assessed the effect of each mediator on the risk of kidney stones (β2) in the UK Biobank. Third, the mediation proportion of each mediator within the comprehensive impact of education on kidney stones was determined by dividing the indirect effect, derived from the combined estimates of the two sequential steps (β1*β2), by the overall effect.

### Statistical analysis

In the study, the following multiple methods were used to evaluate the causal relationship: random-effect inverse variance weighted (IVW), MR-Egger, weighted median, simple mode, and weighted mode. IVW is a meta-analysis method that obtains an overall estimate of the effect of each risk factor on the risk of kidney stones by combining the Wald ratios of each single nucleotide polymorphism (SNP). MR-Egger not only obtains a causal effect estimate but also assesses potential horizontal pleiotropic effects. Weighted median, simple mode, and weighted mode were regarded as supplements to IVW. The MR-PRESSO approach can detect pleiotropic outliers, which are then removed manually. The strength of IVs for each risk factor was evaluated by F statistics (F = beta^2^/se^2^). Cochrane’s Q statistic was used to assess the heterogeneity of IVs. Scatter plots and funnel plots allow the visualization of MR analysis results and potential outliers. Additionally, we performed “Leave-one-out” analysis to identify potentially heterogeneous SNPs. We conducted a meta-analysis using the fixed-effect model to combine the IVW analysis results from the UKBB and FinnGen consortium. All data analysis were completed using R software (version 4.1.3).

## Results

The number of SNPs of risk factors varied from 2 to 449 and the explained phenotypic variances ranged from 0.07% to 7.84%. The general F statistics of each risk factor were greater than 10, suggesting there were no weak IVs biases (**Table 1**).

**Table 1:** Characteristics of the GWAS datasets.

Exposure	Ethnicity	Consortium	Sample size	PMID	FinnGen				UKBB		
					SNPs	F	R^2^(%)		SNPs	F	R^2^(%)
**Lifestyle factors**											
Alcohol quantity (per week)	European	GSCAN	941,280	30643251	35	75.79	0.28		35	74.35	0.28
Smoking initiation	European	GSCAN	1,232,091	30643251	85	26.77	0.28		91	40.83	0.30
Coffee intake	European	Meta-analysis (UKBB；Nurses’ Health Study)	375,833	31046077	11	91.97	0.61		11	209.43	0.61
Plasma caffeine levels	European	Meta-analysis (6 cohorts)	9,876	27702941	2	67.05	1.34		2	67.05	1.34
Carbohydrate intake	European	SSGAC	268,922	32393786	8	35.61	0.11		10	34.85	0.13
Fat intake	European	SSGAC	268,922	32393786	3	62.37	0.07		5	64.14	0.12
Protein intake	European	SSGAC	268,922	32393786	3	44.95	0.05		5	56.76	0.11
Sleep duration	European	Meta-analysis (UKBB; 23andMe)	1,331,010	30846698	54	40.36	0.16		57	41.45	0.18
Insomnia	European	Meta-analysis (UKBB; 23andMe)	1,331,010	30804565	143	43.02	0.46		151	42.59	0.48
Moderate-vigorous physical activity	European	UKBB	377,234	29899525	17	34.29	0.15		18	34.08	0.16
Watching TV (sedentary behavior)	European	UKBB	422,218	32317632	70	48.18	0.80		76	47.40	0.85
Educational attainment	European	Meta-analysis (UKBB; SSGAC; 23andMe)	1,131,881	30038396	408	196.67	7.09		430	54.67	2.08
**Serum and urine parameters**											
Urinary sodium	European	UKBB	446,237	31409800	40	49.03	0.44		43	48.86	0.47
Urinary potassium	European	UKBB	446,237	31409800	12	44.91	0.12		12	44.91	0.12
Urinary sodium/potassium ratio	European	UKBB	478,311	32008434	17	39.41	0.14		21	45.01	0.20
Urinary sodium/creatinine ratio	European	UKBB	478,311	32008434	11	62.40	0.14		13	59.11	0.16
Urinary potassium/creatinine ratio	European	UKBB	478,311	32008434	12	66.10	0.17		14	65.52	0.19
Serum calcium	European	UKBB	325,659	35134646	86	66.98	1.77		89	67.40	1.84
Serum phosphate	European	UKBB	431,448	NA	106	105.48	2.59		115	102.53	2.73
PTH	European	Meta analysis (13 cohorts)	29,155	27927781	3	125.93	1.29		3	125.93	1.29
25OHD	European	UKBB	443,734	32059762	36	345.99	2.77		38	302.07	2.55
Vitamin C	European	Meta analysis (Fenland; EPIC)	52,018	33203707	5	108.72	1.04		7	61.07	0.82
CRP	European	Meta analysis (HapMap; 1KG)	204,402	30388399	37	203.63	3.67		37	202.61	3.65
eGFRcrea	European	Meta analysis (CKDGen; UKBB)	1,004,040	34272381	271	79.25	2.14		284	80.28	2.27
eGFRcys	European	Meta analysis (CKDGen; UKBB)	460,826	34272381	87	69.04	1.29		90	66.31	1.29
BUN	European	Meta analysis (CKDGen; UKBB)	852,678	34272381	50	93.13	0.55		55	88.03	0.57
Urate	European	Meta analysis (UKBB)	454,183	33356394	154	96.30	3.28		159	96.73	3.40
Testosterone	European	UKBB	425,097	32042192	129	139.73	4.23		140	128.76	4.23
Estradiol	European	UKBB	425,097	32042192	10	84.83	0.20		10	87.63	0.21
HDL cholesterol	European	GLGC	188,577	24097068	82	121.28	5.26		81	121.69	5.21
LDL cholesterol	European	GLGC	188,577	24097068	74	163.10	6.38		74	164.98	6.45
Total cholesterol	European	GLGC	188,577	24097068	78	139.42	5.75		81	137.97	5.91
Triglycerides	European	GLGC	188,577	24097068	52	144.32	3.97		52	144.36	3.97
**Metabolic comorbidities**											
BMI	European	Meta analysis (UKBB; GIANT)	681,275	30124842	437	73.06	4.70		449	74.94	4.94
Waist circumference	European	GIANT	224,459	25673412	40	48.51	0.86		42	49.45	0.92
T2DM	European	DIAMANTE	898,130	34088700	140	71.41	1.11		142	71.77	1.13
Fasting glucose	European	MAGIC	188,577	27841877	43	116.26	2.64		43	115.85	2.63
Fasting insulin	European	MAGIC	188,577	27841877	30	48.63	0.77		31	48.03	0.79
Glycated hemoglobin	European	MAGIC	188,577	27841877	172	83.39	7.60		178	83.10	7.84
Hypertension	European	MRC-IEU	461,880	NA	35	54.83	0.42		41	52.80	0.47
DBP	European	Meta-analysis (UKBB; ICBP)	757,601	30224653	381	79.96	4.61		390	78.97	4.68
SBP	European	Meta-analysis (UKBB; ICBP)	757,601	30224653	385	74.08	3.76		395	73.67	3.84
CAD	European	Meta-analysis (UKBB; CARDIoGRAMplusC4D)	547,261	29212778	101	76.68	1.41		107	74.85	1.46
Ischemic stroke	European	MEGASTROKE	446,696	29531354	3	749.09	0.50		3	749.09	0.50

UKBB, UK Biobank; SNP, single nucleotide polymorphisms; GSCAN, GWAS and Sequencing Consortium of Alcohol and Nicotine use; SSGAC, Social Science Genetic Association Consortium; PTH, parathyroid hormone; 25OHD, 25-hydroxyvitamin D; EPIC, European Prospective Investigation into Cancer and Nutrition; CRP, C-reactive protein; 1KG, 1000 Genomes; GFR, glomerular filtration rate; eGFRcrea, GFR estimated by creatinine; eGFRcys, GFR estimated by serum cystatin C; BUN, blood urea nitrogen; CKDGen, Chronic Kidney Disease Genetics Consortium; GLGC, Global Lipids Genetics Consortium; GIANT, Genetic Investigation of ANthropometric Traits; BMI, body mass index; T2DM, Type 2 Diabetes Mellitus; MAGIC, Meta-Analyses of Glucose and Insulin-Related Traits; MRC-IEU, MRC Integrative Epidemiology Unit; DBP, diastolic blood pressure; SBP, systolic blood pressure; ICBP, International Consortium of Blood Pressure Genome Wide Association Studies; CAD, coronary artery disease.

### Discovery results of kidney stones in the FinnGen consortium

In the discovery phase, the IVW method showed that a total of 13 exposures were associated with the risk of kidney stones (P ≤ 0.05) (**Tables S1, S2**). Among them, urinary sodium, urinary potassium, the urinary sodium/creatinine ratio, serum calcium, eGFRcrea, eGFRcys, fasting insulin, and hypertension might increase the risk of kidney stones. Alcohol quantity (per week), coffee intake, plasma caffeine levels, educational attainment, and serum phosphate may decrease the risk of kidney stones. Heterogeneities were detected in the SNPs of the following exposures: alcohol quantity (per week), educational attainment, urinary sodium, the urinary sodium/creatinine ratio, serum calcium, serum phosphate, eGFRcrea, eGFRcys, and hypertension. However, no horizontal pleiotropy was found in the exposures associated with kidney stones.

### Replication results of kidney stones in the UKBB consortium

In the replication phase, the IVW method showed that a total of 19 exposures were associated with the risk of kidney stones (P ≤ 0.05) (**Tables S3, S4**). The following risk factors may increase the risk of kidney stones: smoking initiation, watching TV (sedentary behavior), urinary sodium, the urinary sodium/potassium ratio, the urinary sodium/creatinine ratio, serum calcium, 25OHD, eGFRcrea, eGFRcys, BMI, waist circumference, T2DM, fasting insulin, glycated hemoglobin, and hypertension. Coffee intake, plasma caffeine levels, educational attainment, and the urinary potassium/creatinine ratio may decrease the risk of kidney stones. We observed heterogeneities in the following SNPs of the following exposures: smoking initiation, educational attainment, urinary sodium, the urinary sodium/potassium ratio, serum calcium, serum phosphate, eGFRcrea, eGFRcys, BMI, T2DM, and hypertension. However, we failed to find horizontal pleiotropy in the exposures.

### Combined results of kidney stones from meta-analysis

**Figure 2:**
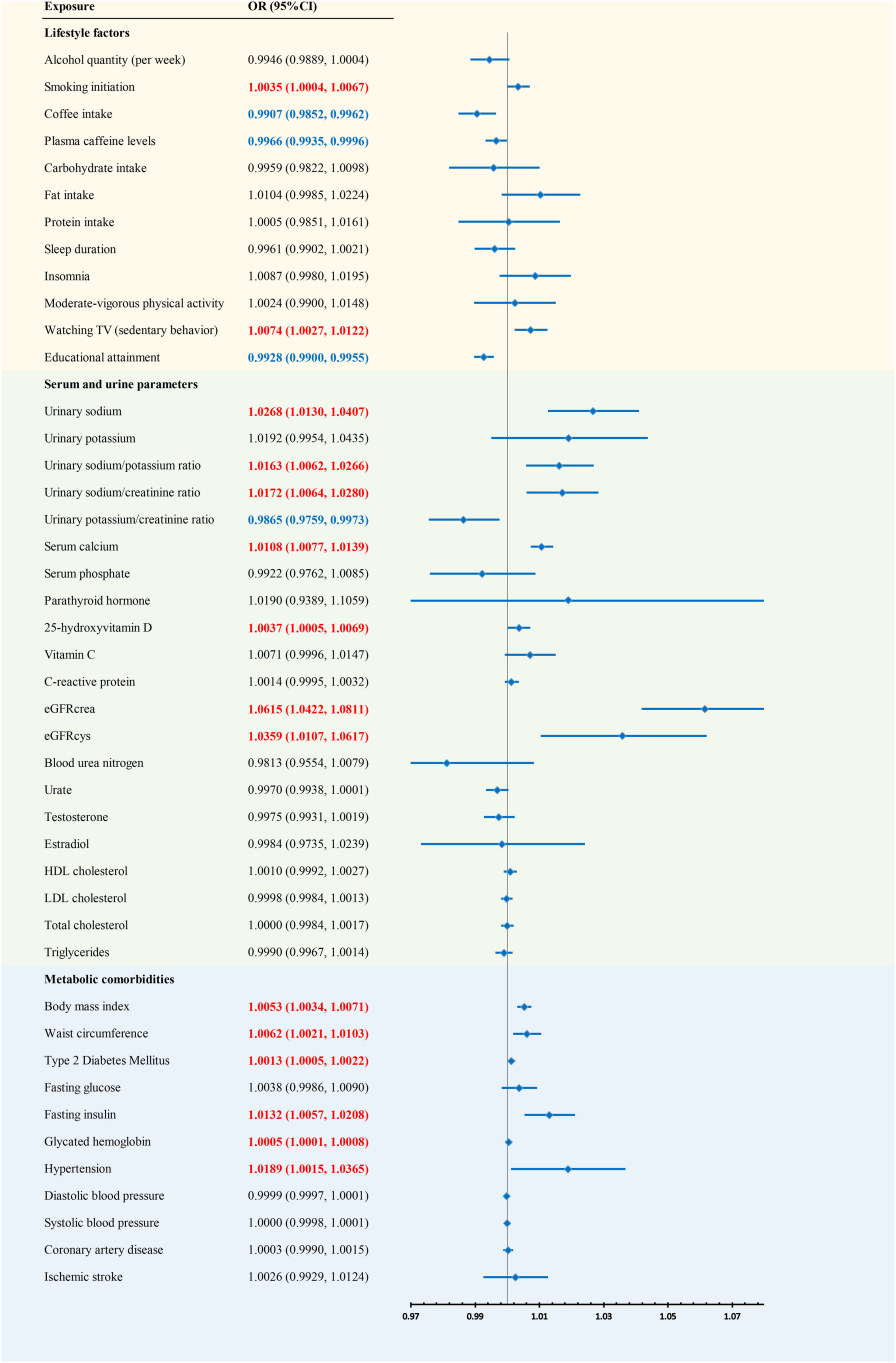
Forest plot of results from meta-analysis.

We conducted a meta-analysis combining the analysis results from the FinnGen and UKBB consortia (**Figure 2**). The combined results indicated that previous exposures may increase the risk of kidney stones, including smoking initiation, watching TV (sedentary behavior), urinary sodium, the urinary sodium/potassium ratio, the urinary sodium/creatinine ratio, serum calcium, 25OHD, eGFRcrea, eGFRcys, BMI, waist circumference, T2DM, fasting insulin, glycated hemoglobin, and hypertension. Coffee intake, plasma caffeine levels, educational attainment, and the urinary potassium/creatinine ratio may decrease the risk of kidney stones.

### Mediation MR analysis

**Tables S5** displays the effects of genetically predicted education and five modifiable risk factors through univariable MR analyses. **Tables S6** displays the causal associations of each mediator with kidney stones with adjustment for education. Each 1-SD increase in education was linked to a reduced BMI (IVW β1: −0.252; 95% CI: −0.327, -0.177), waist circumference (IVW β1: −0.122; 95% CI: −0.167, −0.077), smoking initiation (IVW β1: −0.443; 95% CI: −0.501, −0.385), sedentary behavior (IVW β1: −0.603; 95% CI: −0.636, −0.570), and T2DM (IVW β1: −0.659; 95% CI: −0.771, −0.547). Each 1-SD higher BMI (IVW β2: 0.005; 95% CI: 0.003, 0.007), waist circumference (IVW β2: 0.004; 95% CI: 0.002, 0.006), smoking initiation (IVW β2: 0.004; 95% CI: 0.001, 0.007), sedentary behavior (IVW β2: 0.007; 95% CI: 0.003, 0.010), and T2DM (IVW β2: 0.001; 95% CI: 0.0002, 0.002) were associated with an increased risk of kidney stones after adjusting for education. Ranked by mediation proportion, the effect of education on the risk of kidney stones was mediated by five modifiable risk factors, including sedentary behavior (mediation proportion: 25.7%; 95% CI: 23.3%, 28.1%), smoking initiation (mediation proportion: 10.2%; 95% CI: 7.6%,12.9%), BMI (mediation proportion: 8.2%; 95% CI: 6.1%,10.4%), T2DM (mediation proportion: 5.8%; 95% CI: 0%,13.1%), and waist circumference (mediation proportion: 3.2%; 95% CI: 2.2%,4.1%).

## Discussion

The present MR study analyzed the causal relationship between 44 modifiable risk factors and kidney stones. Using the latest and most extensive kidney stone GWAS, we not only confirmed some of the previous MR studies results, but also found new risk factors associated with kidney stones. Mediation MR analysis showed the causal mediators included sedentary behavior (25.7%), smoking initiation (10.2%), BMI (8.2%), T2DM (5.8%), and waist circumference (3.2%) in the association between education and kidney stones.

### Lifestyle factors

Yuan et al. discovered that higher coffee and caffeine consumption may decrease the risk of kidney stones (51). Although we analyzed the data from the seventh release of the FinnGen consortium and the UKBB, which included more cases and controls, the results were consistent with this. The possible mechanisms are that caffeine has diuretic properties and may reduce the calcium oxalate crystal adhesion of renal tubular epithelial cells (52, 53). After including the most comprehensive GWAS dataset, we observed that lower educational attainment was associated with a higher risk of kidney stones, which is in keeping with the previous study (54). A possible reason is that education could affect kidney stones mediated by a variety of factors, such as smoking, dietary habits, obesity, and so on. We genetically found the associations between smoking initiation and an increased risk of kidney stones. Smoking may increase vasopressin levels, leading to low urine output, which increases the risk of kidney stones (55). Moreover, increased oxidative stress in the kidneys is also a potential mechanism (55). Consistent with a large meta-analysis (56), we failed to discover an association between moderate-vigorous physical activity and kidney stones, which runs counter to many previous studies. However, we observed that sedentary behavior can increase the risk of kidney stones, and whether this is mediated by obesity or not needs to be further investigated.

### Serum and urine parameters

We first genetically discovered that levels of urinary sodium, the urinary sodium/potassium ratio, and the urinary sodium/creatinine ratio were positively associated with the risk of kidney stones. In the 1990s, Cirllo et al. revealed that the urinary sodium/potassium ratio and urinary sodium/creatinine ratio were significantly related to the prevalence of urinary stone diseases (57). High urinary calcium excretion can increase the relative risk of urinary stone formation (58). Many experiments demonstrated the level of urinary calcium excretion increased with urinary sodium excretion and dietary sodium intake in stone formers and healthy individuals (59–61). A low sodium diet may reduce urinary calcium excretion levels and thus it might be an effective approach for preventing urinary stones.

We also found lower levels of eGFRcrea and eGFRcys may decrease the risk of kidney stones, which can be largely explained by renal failure shortening the lifespan of patients, as the risk of kidney stones increases with the age (62), and lower urinary calcium excretion in people with chronic kidney disease than those with normal kidney function (63, 64). A cross-sectional study showed a progressive decrease of urinary calcium excretion with the progression of chronic kidney disease (64). However, our results only suggested a causal relationship between eGFR and kidney stones; it is clearly impossible to prevent urinary stones by reducing renal function. Moreover, we genetically confirmed that higher levels of serum calcium and 25(OH)D were significantly associated with kidney stones (65). Several studies indicated that a high level of vitamin D and vitamin D supplementation can increase hypercalcemia and hypercalciuria (66, 67).

### Metabolic comorbidities

Obesity and T2DM were revealed to be positively associated with kidney stones based on cross-sectional and cohort studies (68, 69), which was consistent with our MR results and others (70). We further discovered that waist circumference, fasting insulin, glycated hemoglobin, and hypertension are also significantly associated with a higher risk of kidney stones. A meta-analysis showed that there were positive non-linear associations between BMI and waist circumference and kidney stones, and the relative risk of kidney stones increased by 21% per 5 kg/m (2) increase in BMI, 16% per 10 cm increase in waist circumference, and 16% among diabetes patients (56). Obesity can increase the risk of kidney stones in multiple ways. Excessive food intake may cause metabolic disorders of calcium, sodium, oxalate, and uric acid (71), which is the reason some obese people are prone to urinary stones. Additionally, fatty acid-binding protein 4 (FABP4), an essential member of the fatty acid-binding protein family (72), was downregulated in collecting duct epithelial cells in renal papillae with Randall plaques, and FABP4 knockout mice developed both interstitial calcium and renal tubular crystals (73). Moreover, the chronic inflammatory state in obesity plays an essential role in the development of kidney stones. Weinberg et al. investigated associations between diabetic severity and the risk of kidney stones and found that a history of T2DM, fasting plasma insulin, and glycosylated hemoglobin A_1_c was significantly associated with kidney stones even after adjusting for potential confounders (74). Insulin resistance may induce derangements of urine pH and the renal handling of calcium and ammonium, which can largely explain the increased risk of urinary stones for diabetics. Some studies have indicated the diabetics have increased urinary calcium and phosphorus excretion, and others have shown an increased urinary oxalate excretion in patients with kidney stones and T2DM (75, 76). Additionally, studies have demonstrated increased uric acid excretion in diabetics, which may be a mechanism in which uric acid is the main component of urinary stones in this instance (77). Furthermore, obesity is associated with insulin resistance, which can promote urinary stone formation. Hypercalciuria may be the pathogenetic factor of kidney stones in patients with hypertension (78).

Although the analytical outcomes of the two GWAS databases largely converge, disparities are observed in the causal relationships between alcohol quantity, smoking initiation, sedentary behavior, educational attainment, urinary potassium, 25OHD, BMI, waist circumference, T2DM, and kidney stone disease. These distinctions may be attributed to factors such as population disparities, research methodologies, lifestyle patterns, and educational levels. In terms of population differences, the Finnish database primarily consists of individuals of Finnish descent, representing a homogenous Northern European population with distinct genetic characteristics. On the other hand, the UK Biobank database encompasses a much more diverse range of ethnic backgrounds due to the multicultural nature of the UK, encompassing various ethnicities such as Caucasian, Asian, and African. Regarding analytical methods, the Finnish database might have tailored statistical approaches to accommodate the genetic makeup of the Finnish population. By contrast, the UK Biobank would have likely employed diverse analytical strategies to consider the multitude of ethnic groups represented in the dataset. Disparities in lifestyle patterns and education levels are evident. Finland’s lifestyle habits, dietary preferences, and exercise routines may be influenced by local customs and Northern European cultural norms. Meanwhile, the UK Biobank database reflects the diverse lifestyle practices and dietary choices associated with the various ethnic groups residing in the UK.

### Mediation MR analysis

Educational attainment is closely intertwined with human health. We used a two-step MR method to identify causal mediators that significantly elucidate the causal relationship between education and the occurrence of kidney stones. These findings align with the primary mechanisms underlying kidney stone development, in which sedentary habits, obesity, smoking, and T2DM emerge as pivotal risk factors for kidney stone formation (9, 22, 23). Low educational attainment increases susceptibility to kidney stone diseases, potentially by reducing access to economic, cultural, and social resources. These reductions may contribute to risk factors such as smoking, body weight, and some metabolic diseases (79, 80).

### Strengths and limitations

Our study has several strengths. First, the MR design is suitable for exploring the causal relationships in the presence of potential confounders and reverse causation. Second, we used the latest and most extensive kidney stones GWAS and genetically found quite a few new risk factors associated with kidney stones, such as smoking initiation, sedentary behavior, urinary sodium, the urinary sodium/potassium ratio, the urinary sodium/creatinine ratio, waist circumference, fasting insulin, glycated hemoglobin, and hypertension. Third, our study included discovery, validation, and meta-analysis stages, enhancing the causal relationship between exposures and kidney stones. Finally, the study population was entirely of European ancestry, which reduced population stratification bias.

However, there are some limitations in our MR study. The biggest challenge is possible horizontal pleiotropy, meaning genetic variants influence the risk of kidney stones not via the exposure that we are studying. MR-Egger intercept was applied to detect the horizontal pleiotropy, and most of the results were stable. In addition, the heterogeneity of some IVs was not avoided due to the differences in study type, subgroup population, region, and so on. Finally, our study was restricted to European populations and the results may not be generalizable to other ethnic populations.

## Conclusions

Our MR study shows causal associations of earlier smoking initiation, increased sedentary behavior, urinary sodium, the urinary sodium/potassium ratio, the urinary sodium/creatinine ratio, serum calcium, 25OHD, eGFRcrea, eGFRcys, BMI, waist circumference, fasting insulin, glycated hemoglobin, a history of T2DM, and hypertension with an increased risk of kidney stones. Increased coffee intake, plasma caffeine levels, educational attainment, and the urinary potassium/creatinine ratio are associated with a decreased risk of kidney stones. Mediation MR analysis expounded upon the causal impacts of educational attainment on kidney stones and quantified distinct causal mediators within relevant pathways, including sedentary lifestyles, obesity, smoking, and T2DM. Our results suggest more attention should be paid to these modifiable factors to prevent kidney stones.

## Data availability statement

The original contributions presented in the study are included in the article/**Supplementary Material**. Further inquiries can be directed to the corresponding authors.

## Ethics statement

The Mendelian randomization studies conducted using the UK Biobank data might not require ethical review. The studies were conducted in accordance with the local legislation and institutional requirements. The participants provided their written informed consent to participate in this study.

## Author contributions

ML: project development, data analysis, and manuscript writing. JW and MG: data collection and analysis. WX: data collection. JC and ZC: manuscript editing. ZZ and HC: project development and manuscript editing. All authors contributed to the article and approved the submitted version.

## Supplementary material

Supplementary Figure 1 Scatter plots for the causal association between 44 modifiable risk factors and kidney stones in the FinnGen consortium. (TIF)

Supplementary Figure 2 Scatter plots for the causal association between 44 modifiable risk factors and kidney stones in the UK Biobank consortium. (TIF)

Supplementary Figure 3 Funnel plots for the causal association between 44 modifiable risk factors and kidney stones in the FinnGen consortium. (TIF)

Supplementary Figure 4 Funnel plots for the causal association between 44 modifiable risk factors and kidney stones in the UK Biobank consortium. (TIF)

Supplementary Figure 5 Leave-one-out plots for the causal association between 44 modifiable risk factors and kidney stones in the FinnGen consortium. (TIF)

Supplementary Figure 6 Leave-one-out plots for the causal association between 44 modifiable risk factors and kidney stones in the UK Biobank consortium. (TIF)
