# Supplementary figures and images for: Lifestyle factors, serum parameters, metabolic comorbidities, and the risk of kidney stones: a Mendelian randomization study
Source: Front Endocrinol (Lausanne). 2023 Sep 22;14:1240171. doi: 10.3389/fendo.2023.1240171 (PMC10560039; doi:10.3389/fendo.2023.1240171)

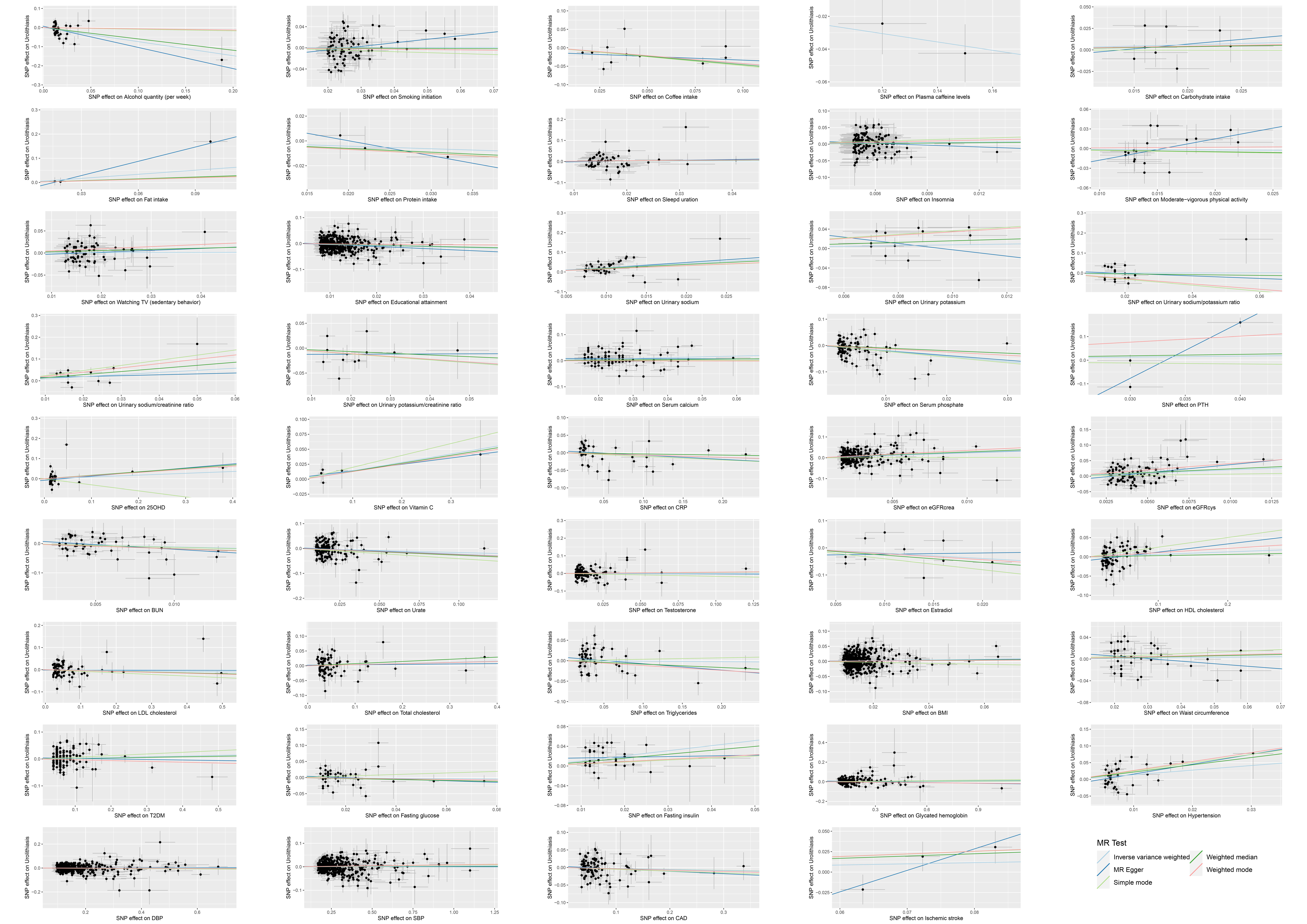

Supplement: Supplementary Figure 1 — Scatter plots for the causal association between 44 modifiable risk factors and kidney stones in the FinnGen consortium. (TIF) [file Image_1.tif]

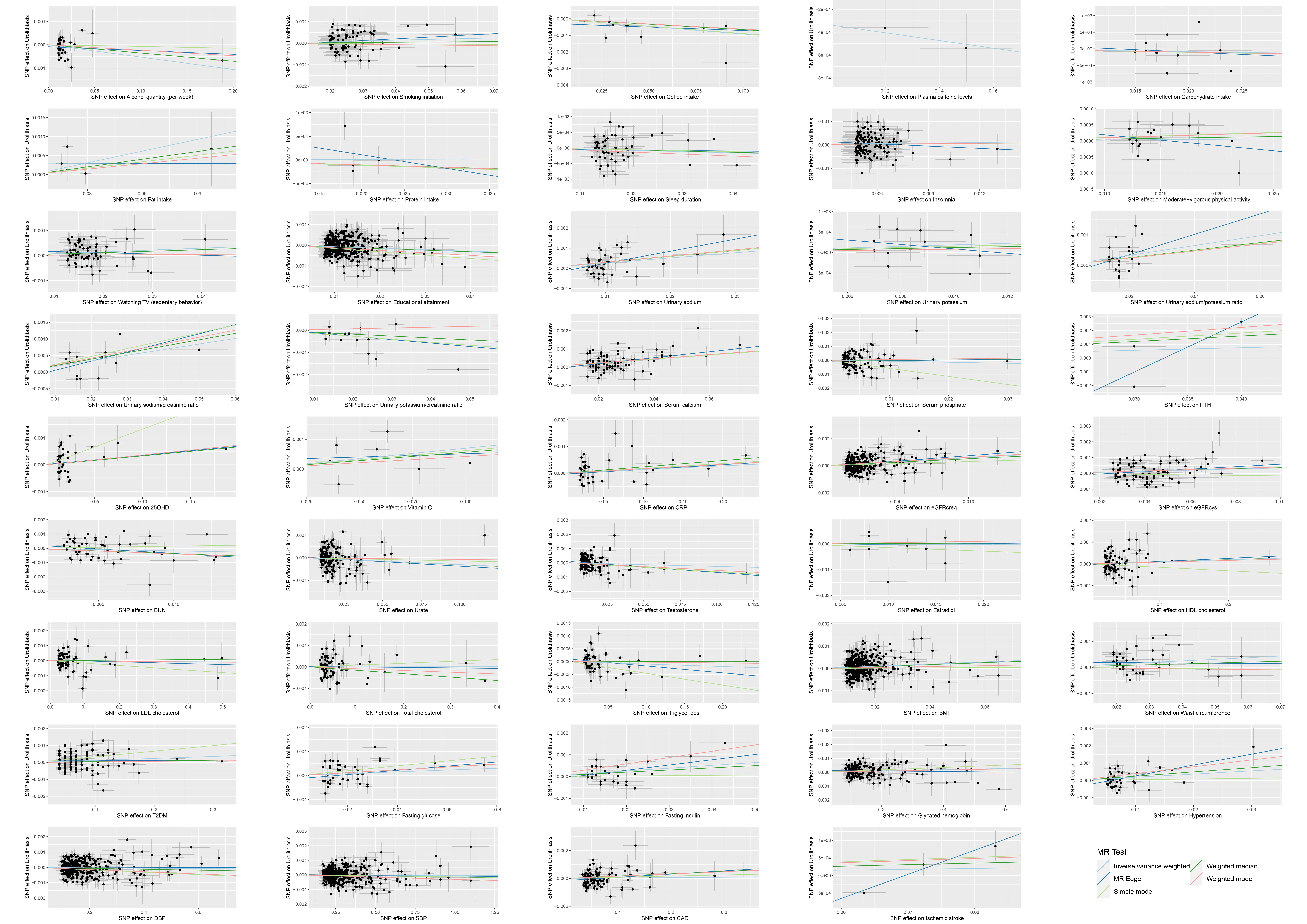

Supplement: Supplementary Figure 2 — Scatter plots for the causal association between 44 modifiable risk factors and kidney stones in the UK Biobank consortium. (TIF) [file Image_2.tif]

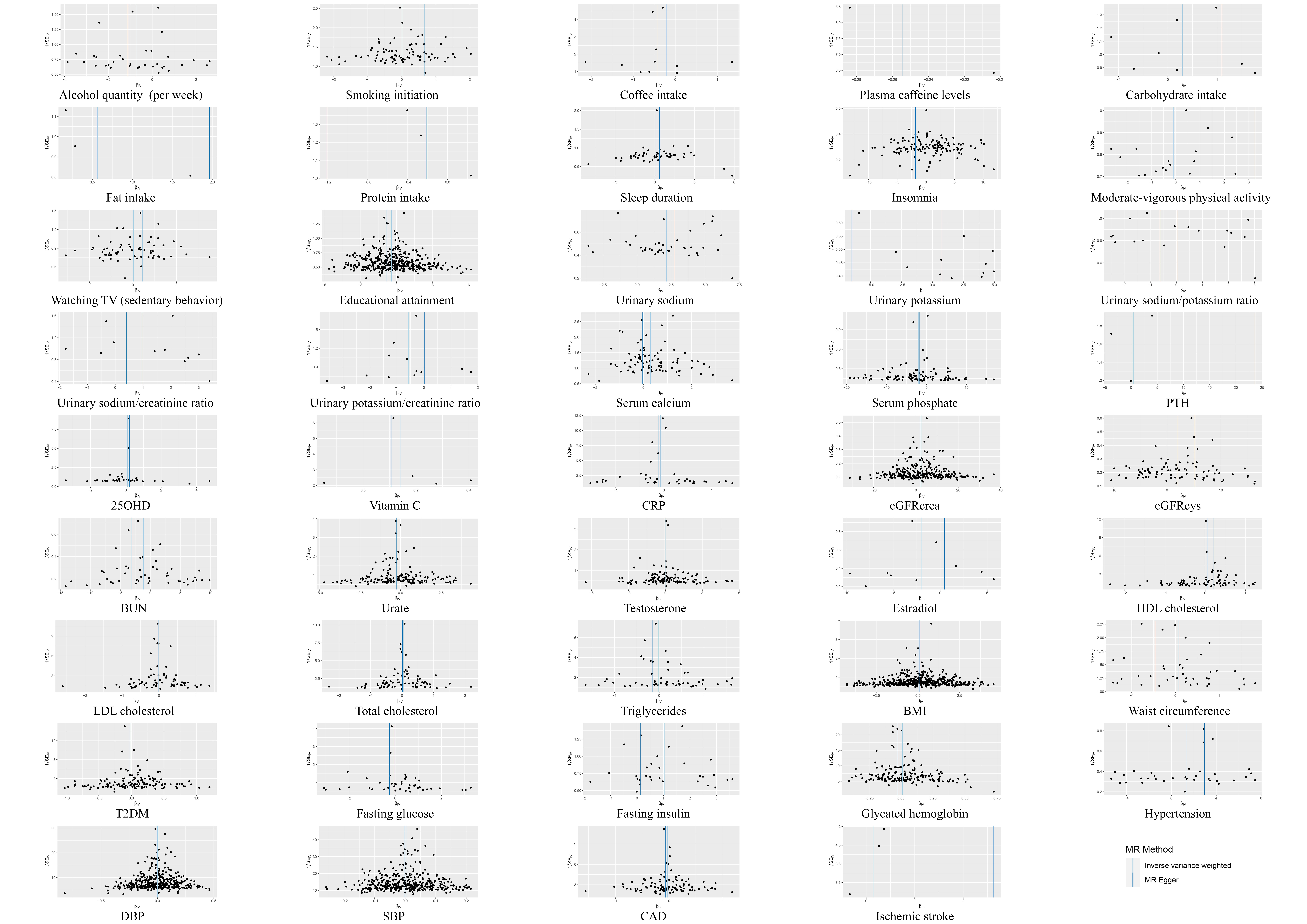

Supplement: Supplementary Figure 3 — Funnel plots for the causal association between 44 modifiable risk factors and kidney stones in the FinnGen consortium. (TIF) [file Image_3.tif]

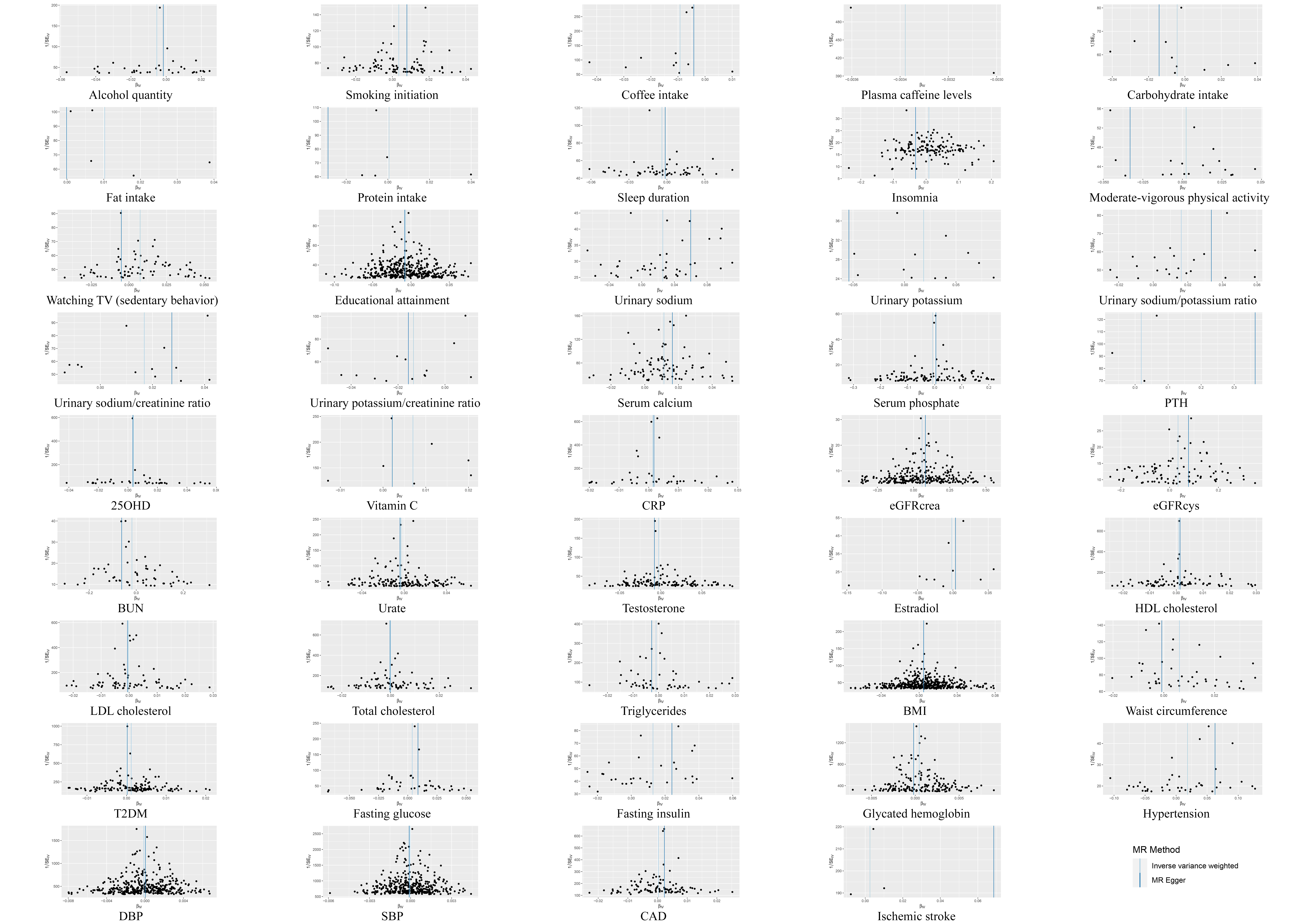

Supplement: Supplementary Figure 4 — Funnel plots for the causal association between 44 modifiable risk factors and kidney stones in the UK Biobank consortium. (TIF) [file Image_4.tif]
