# Supplementary material for: Lifestyle factors, serum parameters, metabolic comorbidities, and the risk of kidney stones: a Mendelian randomization study
Source: Front Endocrinol (Lausanne). 2023 Sep 22;14:1240171. doi: 10.3389/fendo.2023.1240171 (PMC10560039; doi:10.3389/fendo.2023.1240171)
Supplement: Supplementary file 7 [file Table_1.docx]

Table S1 Causal association of potentially modifiable risk factors on kidney stones in FinnGen consortium.

| **Exposure** | **IVW** | |  | **MR-Egger** | |  | **Weighted median** | |  | **Simple mode** | |  | **Weighted mode** | | **MR-PRESSO Global** | **Heterogeneity** | **Pleiotropy** |
| --- | --- | --- | --- | --- | --- | --- | --- | --- | --- | --- | --- | --- | --- | --- | --- | --- | --- |
|  | **OR (95%CI)** | ***P*** |  | **OR (95%CI)** | ***P*** |  | **OR (95%CI)** | ***P*** |  | **OR (95%CI)** | ***P*** |  | **OR (95%CI)** | ***P*** | ***P*** | ***P*** | ***P*** |
| **Lifestyle factors** | | | | | | | | | | | | | | | | | |
| Alcohol quantity (per week) | 0.479 (0.290,0.793) | 0.004 |  | 0.331 (0.103,1.061) | 0.072 |  | 0.553 (0.286,1.070) | 0.078 |  | 0.919 (0.189,4.470) | 0.918 |  | 0.939 (0.281,3.137) | 0.919 | 0.021 | 0.022 | 0.493 |
| Smoking initiation | 1.009 (0.849,1.198) | 0.922 |  | 1.964 (0.868,4.442) | 0.109 |  | 0.978 (0.770,1.242) | 0.853 |  | 0.834 (0.478,1.453) | 0.523 |  | 0.937 (0.583,1.505) | 0.788 | 0.029 | 0.083 | 0.106 |
| Coffee intake | 0.648 (0.465,0.901) | 0.010 |  | 0.816 (0.421,1.582) | 0.563 |  | 0.631 (0.457,0.870) | 0.005 |  | 0.615 (0.374,1.010) | 0.084 |  | 0.653 (0.478,0.891) | 0.023 | 0.099 | 0.069 | 0.446 |
| Plasma caffeine levels | 0.775 (0.645,0.932) | 0.007 |  | NA | NA |  | NA | NA |  | NA | NA |  | NA | NA | NA | 0.682 | NA |
| Carbohydrate intake | 1.345 (0.683,2.646) | 0.391 |  | 2.998 (0.038,237.753) | 0.640 |  | 1.204 (0.497,2.916) | 0.682 |  | 0.983 (0.221,4.376) | 0.983 |  | 1.240 (0.299,5.141) | 0.776 | 0.313 | 0.385 | 0.728 |
| Fat intake | 1.755 (0.548,5.621) | 0.343 |  | 7.135 (0.386,131.730) | 0.413 |  | 1.284 (0.314,5.258) | 0.728 |  | 1.253 (0.226,6.940) | 0.820 |  | 1.236 (0.259,5.892) | 0.815 | NA | 0.561 | 0.491 |
| Protein intake | 0.810 (0.320,2.050) | 0.657 |  | 0.299 (0.004,24.319) | 0.686 |  | 0.739 (0.268,2.036) | 0.559 |  | 0.715 (0.207,2.477) | 0.650 |  | 0.709 (0.212,2.366) | 0.632 | NA | 0.870 | 0.728 |
| Sleep duration | 1.081 (0.728,1.603) | 0.700 |  | 1.431 (0.308,6.644) | 0.649 |  | 1.163 (0.693,1.949) | 0.568 |  | 1.161 (0.360,3.742) | 0.804 |  | 1.194 (0.554,2.576) | 0.653 | 0.023 | 0.009 | 0.712 |
| Insomnia | 1.686 (0.866,3.284) | 0.124 |  | 0.163 (0.002,11.220) | 0.402 |  | 1.431 (0.611,3.353) | 0.409 |  | 4.441 (0.421,46.861) | 0.217 |  | 2.804 (0.311,25.276) | 0.360 | 0.001 | <0.001 | 0.275 |
| Moderate-vigorous physical activity | 0.908 (0.449,1.839) | 0.789 |  | 26.057 (0.638,1063.898) | 0.105 |  | 0.787 (0.325,1.908) | 0.597 |  | 0.761 (0.119,4.856) | 0.776 |  | 1.107 (0.230,5.334) | 0.901 | 0.136 | 0.142 | 0.092 |
| Watching TV (sedentary behavior) | 1.031 (0.788,1.351) | 0.822 |  | 1.537 (0.432,5.475) | 0.509 |  | 1.316 (0.889,1.947) | 0.170 |  | 1.618 (0.577,4.539) | 0.364 |  | 1.618 (0.674,3.885) | 0.286 | 0.131 | 0.379 | 0.531 |
| Educational attainment | 0.666 (0.560,0.793) | <0.001 |  | 0.450 (0.237,0.855) | 0.015 |  | 0.707 (0.550,0.910) | 0.007 |  | 0.884 (0.378,2.068) | 0.777 |  | 0.884 (0.442,1.770) | 0.729 | 0.004 | 0.001 | 0.214 |
| **Serum and urine parameters** | | | | | | | | | | | | | | | | | |
| Urinary sodium | 8.628 (3.898,19.101) | <0.001 |  | 15.080 (0.370,615.358) | 0.160 |  | 6.845 (2.466,19.003) | <0.001 |  | 4.925 (0.460,52.683) | 0.195 |  | 5.089 (0.234,110.670) | 0.307 | 0.006 | 0.005 | 0.764 |
| Urinary potassium | 7.361 (1.411,38.392) | 0.018 |  | 474.099 (0.002,>10E+7) | 0.349 |  | 10.589 (1.643,68.222) | 0.013 |  | 49.784 (1.844,1343.750) | 0.042 |  | 40.789 (1.411,1179.121) | 0.056 | 0.023 | 0.110 | 0.517 |
| Urinary sodium/potassium ratio | 1.023 (0.414,2.530) | 0.960 |  | 0.533 (0.002,125.164) | 0.824 |  | 0.832 (0.327,2.116) | 0.699 |  | 0.225 (0.022,2.326) | 0.229 |  | 0.263 (0.034,2.040) | 0.220 | 0.001 | <0.001 | 0.815 |
| Urinary sodium/creatinine ratio | 2.611 (1.053,6.478) | 0.038 |  | 1.503 (0.048,47.133) | 0.822 |  | 4.131 (1.551,11.003) | 0.005 |  | 10.543 (1.544,71.979) | 0.037 |  | 7.207 (0.989,52.506) | 0.080 | 0.006 | 0.004 | 0.751 |
| Urinary potassium/creatinine ratio | 0.567 (0.298,1.082) | 0.085 |  | 1.026 (0.113,9.284) | 0.982 |  | 0.710 (0.317,1.587) | 0.404 |  | 0.555 (0.175,1.757) | 0.338 |  | 0.574 (0.222,1.482) | 0.276 | 0.436 | 0.227 | 0.592 |
| Serum calcium | 1.343 (1.108,1.627) | 0.003 |  | 0.968 (0.508,1.843) | 0.921 |  | 1.114 (0.856,1.450) | 0.421 |  | 1.012 (0.553,1.853) | 0.969 |  | 0.982 (0.505,1.907) | 0.957 | 0.009 | 0.007 | 0.299 |
| Serum phosphate | 0.129 (0.050,0.333) | <0.001 |  | 0.163 (0.035,0.751) | 0.022 |  | 0.378 (0.088,1.619) | 0.190 |  | 0.111 (0.003,3.537) | 0.216 |  | 0.290 (0.080,1.049) | 0.062 | <0.001 | <0.001 | 0.706 |
| PTH | 1.541 (0.012,200.444) | 0.862 |  | NA | NA |  | 1.854 (0.463,7.420) | 0.383 |  | 0.672 (0.020,23.086) | 0.846 |  | NA | NA | NA | <0.001 | 0.240 |
| 25OHD | 1.094 (0.909,1.317) | 0.341 |  | 1.225 (0.986,1.522) | 0.076 |  | 1.186 (0.963,1.461) | 0.108 |  | 0.741 (0.372,1.473) | 0.398 |  | 1.169 (0.961,1.422) | 0.127 | 0.128 | 0.200 | 0.080 |
| Vitamin C | 1.182 (0.840,1.664) | 0.337 |  | 1.091 (0.295,4.029) | 0.905 |  | 1.181 (0.801,1.742) | 0.401 |  | 1.222 (0.714,2.092) | 0.505 |  | 1.181 (0.699,1.997) | 0.567 | 0.964 | 0.932 | 0.908 |
| CRP | 0.934 (0.840,1.039) | 0.208 |  | 0.889 (0.765,1.033) | 0.134 |  | 0.968 (0.849,1.103) | 0.624 |  | 0.903 (0.668,1.221) | 0.512 |  | 0.937 (0.841,1.045) | 0.249 | 0.073 | 0.062 | 0.366 |
| eGFRcrea | 12.041 (4.228,34.292) | <0.001 |  | 10.713 (0.909,126.280) | 0.061 |  | 16.575 (3.967,69.259) | <0.001 |  | 1.153 (0.009,143.295) | 0.954 |  | 34.931 (2.307,528.892) | 0.011 | <0.001 | <0.001 | 0.918 |
| eGFRcys | 7.600 (2.478,23.307) | <0.001 |  | 179.392 (6.503,4948.607) | 0.003 |  | 10.629 (2.411,46.848) | 0.002 |  | 1.936 (0.025,151.908) | 0.767 |  | 58.247 (3.403,997.038) | 0.006 | <0.001 | 0.001 | 0.051 |
| BUN | 0.315 (0.098,1.018) | 0.054 |  | 0.041 (0.003,0.576) | 0.022 |  | 0.176 (0.035,0.891) | 0.036 |  | 0.282 (0.008,9.704) | 0.486 |  | 0.168 (0.026,1.085) | 0.067 | 0.018 | 0.021 | 0.099 |
| Urate | 0.850 (0.702,1.028) | 0.094 |  | 0.772 (0.529,1.127) | 0.182 |  | 0.763 (0.590,0.988) | 0.040 |  | 0.665 (0.379,1.164) | 0.155 |  | 0.782 (0.588,1.039) | 0.092 | <0.001 | <0.001 | 0.567 |
| Testosterone | 0.990 (0.773,1.267) | 0.937 |  | 0.966 (0.631,1.479) | 0.875 |  | 1.067 (0.714,1.595) | 0.752 |  | 0.848 (0.369,1.950) | 0.699 |  | 1.073 (0.736,1.564) | 0.716 | 0.007 | 0.143 | 0.891 |
| Estradiol | 0.141 (0.015,1.336) | 0.088 |  | 1.584 (0.003,719.629) | 0.887 |  | 0.068 (0.009,0.498) | 0.008 |  | 0.018 (0.000,0.698) | 0.060 |  | 0.113 (0.015,0.848) | 0.063 | 0.026 | 0.002 | 0.428 |
| HDL cholesterol | 1.067 (0.968,1.176) | 0.191 |  | 1.239 (1.060,1.449) | 0.009 |  | 1.032 (0.897,1.187) | 0.658 |  | 1.287 (0.956,1.731) | 0.100 |  | 1.117 (0.975,1.280) | 0.114 | 0.075 | 0.067 | 0.021 |
| LDL cholesterol | 0.982 (0.905,1.065) | 0.655 |  | 0.998 (0.889,1.120) | 0.970 |  | 0.964 (0.858,1.083) | 0.541 |  | 0.931 (0.740,1.172) | 0.544 |  | 0.961 (0.863,1.070) | 0.474 | 0.107 | 0.088 | 0.701 |
| Total cholesterol | 1.040 (0.946,1.143) | 0.418 |  | 1.014 (0.872,1.181) | 0.853 |  | 1.076 (0.950,1.218) | 0.249 |  | 1.037 (0.829,1.297) | 0.753 |  | 1.037 (0.933,1.152) | 0.504 | 0.027 | 0.014 | 0.683 |
| Triglycerides | 0.988 (0.859,1.137) | 0.870 |  | 0.856 (0.678,1.080) | 0.197 |  | 0.920 (0.758,1.117) | 0.400 |  | 1.036 (0.701,1.530) | 0.860 |  | 0.889 (0.745,1.060) | 0.194 | 0.014 | 0.010 | 0.138 |
| **Metabolic comorbidities** | | | | | | | | | | | | | | | | | |
| BMI | 1.128 (0.992,1.283) | 0.066 |  | 1.083 (0.769,1.525) | 0.648 |  | 1.082 (0.891,1.314) | 0.427 |  | 0.851 (0.439,1.649) | 0.633 |  | 1.062 (0.603,1.872) | 0.835 | <0.001 | <0.001 | 0.801 |
| Waist circumference | 1.066 (0.833,1.363) | 0.613 |  | 0.627 (0.266,1.475) | 0.291 |  | 1.128 (0.819,1.553) | 0.460 |  | 1.282 (0.607,2.709) | 0.519 |  | 1.155 (0.620,2.153) | 0.653 | 0.152 | 0.107 | 0.212 |
| T2DM | 1.027 (0.974,1.083) | 0.326 |  | 0.981 (0.880,1.102) | 0.729 |  | 1.020 (0.944,1.102) | 0.614 |  | 1.064 (0.887,1.276) | 0.507 |  | 0.970 (0.883,1.067) | 0.533 | 0.002 | 0.003 | 0.346 |
| Fasting glucose | 0.945 (0.710,1.259) | 0.700 |  | 0.779 (0.460,1.320) | 0.359 |  | 0.862 (0.576,1.291) | 0.472 |  | 1.270 (0.592,2.722) | 0.543 |  | 0.907 (0.619,1.328) | 0.617 | 0.145 | 0.166 | 0.397 |
| Fasting insulin | 2.808 (1.809,4.357) | <0.001 |  | 1.150 (0.255,5.194) | 0.857 |  | 2.218 (1.152,4.268) | 0.017 |  | 1.497 (0.422,5.312) | 0.537 |  | 1.576 (0.470,5.286) | 0.467 | 0.497 | 0.457 | 0.235 |
| Glycated hemoglobin | 1.011 (0.988,1.035) | 0.334 |  | 0.976 (0.929,1.025) | 0.327 |  | 1.008 (0.977,1.040) | 0.625 |  | 1.019 (0.938,1.107) | 0.660 |  | 0.984 (0.937,1.034) | 0.524 | <0.001 | <0.001 | 0.106 |
| Hypertension | 3.975 (1.342,11.772) | 0.013 |  | 18.986 (1.196,301.509) | 0.045 |  | 8.830 (2.332,33.433) | 0.001 |  | 12.077 (0.439,332.423) | 0.150 |  | 14.418 (1.793,115.944) | 0.017 | <0.001 | 0.002 | 0.237 |
| DBP | 1.000 (0.987,1.014) | 0.951 |  | 1.007 (0.973,1.041) | 0.705 |  | 0.997 (0.979,1.016) | 0.765 |  | 0.988 (0.934,1.045) | 0.663 |  | 0.994 (0.955,1.033) | 0.746 | <0.001 | <0.001 | 0.698 |
| SBP | 1.003 (0.996,1.011) | 0.398 |  | 0.999 (0.979,1.019) | 0.908 |  | 1.002 (0.991,1.013) | 0.709 |  | 1.008 (0.972,1.045) | 0.686 |  | 1.009 (0.987,1.031) | 0.442 | <0.001 | <0.001 | 0.636 |
| CAD | 0.972 (0.909,1.038) | 0.393 |  | 0.933 (0.812,1.073) | 0.335 |  | 0.972 (0.887,1.066) | 0.549 |  | 0.950 (0.792,1.139) | 0.579 |  | 0.963 (0.861,1.077) | 0.508 | 0.005 | 0.040 | 0.523 |
| Ischemic stroke | 1.155 (0.770,1.732) | 0.487 |  | 13.855 (0.943,203.457) | 0.306 |  | 1.323 (0.924,1.894) | 0.126 |  | 1.371 (0.845,2.223) | 0.329 |  | 1.374 (0.905,2.087) | 0.274 | NA | 0.413 | 0.319 |

**IVW**, inverse variance weighted; **PTH**, parathyroid hormone; **25OHD**, 25-hydroxyvitamin D; **CRP**, C-reactive protein; **GFR**, glomerular filtration rate; **eGFRcrea**, GFR estimated by creatinine; **eGFRcys**, GFR estimated by serum cystatin C; **BUN**, blood urea nitrogen; **BMI**, body mass index; **T2DM**, Type 2 Diabetes Mellitus; **DBP**, diastolic blood pressure; **SBP**, systolic blood pressure; **CAD**, coronary artery disease.
