# Supplementary material for: Lifestyle factors, serum parameters, metabolic comorbidities, and the risk of kidney stones: a Mendelian randomization study
Source: Front Endocrinol (Lausanne). 2023 Sep 22;14:1240171. doi: 10.3389/fendo.2023.1240171 (PMC10560039; doi:10.3389/fendo.2023.1240171)
Supplement: Supplementary file 9 [file Table_3.docx]

Table S3 Causal association of potentially modifiable risk factors on kidney stones in UK Biobank consortium.

| **Exposure** | **IVW** | |  | **MR-Egger** | |  | **Weighted median** | |  | **Simple mode** | |  | **Weighted mode** | | **MR-PRESSO Global** | **Heterogeneity** | **Pleiotropy** |
| --- | --- | --- | --- | --- | --- | --- | --- | --- | --- | --- | --- | --- | --- | --- | --- | --- | --- |
|  | **OR (95%CI)** | ***P*** |  | **OR (95%CI)** | ***P*** |  | **OR (95%CI)** | ***P*** |  | **OR (95%CI)** | ***P*** |  | **OR (95%CI)** | ***P*** | ***P*** | ***P*** | ***P*** |
| **Lifestyle factors** | | | | | | | | | | | | | | | | | |
| Alcohol quantity (per week) | 0.995 (0.989 ,1.001) | 0.074 |  | 0.998 (0.989,1.008) | 0.750 |  | 0.996 (0.988,1.005) | 0.438 |  | 0.999 (0.983,1.016) | 0.932 |  | 0.998 (0.988,1.007) | 0.625 | 0.699 | 0.666 | 0.368 |
| Smoking initiation | 1.004 (1.000,1.007) | 0.028 |  | 1.008 (0.992,1.024) | 0.324 |  | 1.001 (0.997,1.005) | 0.593 |  | 0.999 (0.986,1.012) | 0.903 |  | 0.998 (0.986,1.011) | 0.797 | 0.013 | <0.001 | 0.575 |
| Coffee intake | 0.991 (0.985,0.996) | 0.001 |  | 0.996 (0.985,1.007) | 0.473 |  | 0.993 (0.988,0.999) | 0.014 |  | 0.991 (0.982,1.001) | 0.094 |  | 0.994 (0.989,0.999) | 0.037 | 0.089 | 0.062 | 0.314 |
| Plasma caffeine levels | 0.997 (0.994,1.000) | 0.031 |  | NA | NA |  | NA | NA |  | NA | NA |  | NA | NA | NA | 0.854 | NA |
| Carbohydrate intake | 0.996 (0.982,1.010) | 0.547 |  | 0.986 (0.895,1.086) | 0.782 |  | 0.995 (0.980,1.010) | 0.525 |  | 0.995 (0.973,1.017) | 0.656 |  | 0.995 (0.973,1.017) | 0.669 | 0.121 | 0.054 | 0.844 |
| Fat intake | 1.010 (0.998,1.022) | 0.089 |  | 1.000 (0.975,1.025) | 0.994 |  | 1.007 (0.993,1.021) | 0.339 |  | 1.006 (0.987,1.025) | 0.591 |  | 1.005 (0.989,1.021) | 0.588 | 0.409 | 0.326 | 0.424 |
| Protein intake | 1.001 (0.985,1.016) | 0.943 |  | 0.972 (0.906,1.042) | 0.478 |  | 0.995 (0.981,1.009) | 0.481 |  | 0.994 (0.978,1.011) | 0.546 |  | 0.995 (0.980,1.010) | 0.533 | 0.208 | 0.131 | 0.460 |
| Sleep duration | 0.996 (0.990,1.002) | 0.200 |  | 0.999 (0.977,1.021) | 0.905 |  | 0.997 (0.989,1.004) | 0.371 |  | 0.997 (0.978,1.016) | 0.768 |  | 0.993 (0.979,1.008) | 0.396 | 0.071 | 0.039 | 0.815 |
| Insomnia | 1.009 (0.998,1.019) | 0.116 |  | 0.968 (0.904,1.037) | 0.361 |  | 1.005 (0.992,1.019) | 0.449 |  | 1.005 (0.965,1.048) | 0.800 |  | 1.005 (0.965,1.048) | 0.798 | <0.001 | <0.001 | 0.242 |
| Moderate-vigorous physical activity | 1.002 (0.990,1.015) | 0.705 |  | 0.967 (0.898,1.042) | 0.395 |  | 1.005 (0.991,1.021) | 0.476 |  | 1.011 (0.985,1.037) | 0.441 |  | 1.010 (0.985,1.035) | 0.446 | 0.118 | 0.104 | 0.355 |
| Watching TV (sedentary behavior) | 1.007 (1.003,1.012) | 0.002 |  | 0.995 (0.973,1.018) | 0.662 |  | 1.006 (0.999,1.012) | 0.075 |  | 1.001 (0.984,1.018) | 0.897 |  | 1.001 (0.986,1.017) | 0.885 | 0.06 | 0.151 | 0.273 |
| Educational attainment | 0.993 (0.990,0.996) | <0.001 |  | 0.992 (0.982,1.002) | 0.099 |  | 0.992 (0.988,0.996) | <0.001 |  | 0.984 (0.969,1.000) | 0.047 |  | 0.988 (0.976,0.999) | 0.041 | 0.005 | 0.047 | 0.820 |
| **Serum and urine parameters** | | | | | | | | | | | | | | | | | |
| Urinary sodium | 1.026 (1.012,1.040) | <0.001 |  | 1.062 (1.004,1.123) | 0.043 |  | 1.030 (1.014,1.046) | <0.001 |  | 1.030 (0.989,1.072) | 0.158 |  | 1.031 (0.986,1.078) | 0.186 | 0.001 | <0.001 | 0.228 |
| Urinary potassium | 1.019 (0.995,1.043) | 0.122 |  | 0.947 (0.820,1.095) | 0.482 |  | 1.012 (0.982,1.043) | 0.436 |  | 1.015 (0.962,1.071) | 0.588 |  | 1.008 (0.957,1.061) | 0.771 | 0.050 | 0.181 | 0.343 |
| Urinary sodium/potassium ratio | 1.016 (1.006,1.027) | 0.002 |  | 1.034 (0.990,1.080) | 0.145 |  | 1.013 (1.000,1.025) | 0.044 |  | 1.012 (0.987,1.038) | 0.348 |  | 1.012 (0.986,1.038) | 0.379 | 0.047 | 0.043 | 0.428 |
| Urinary sodium/creatinine ratio | 1.017 (1.006,1.028) | 0.002 |  | 1.028 (0.991,1.066) | 0.172 |  | 1.020 (1.007,1.033) | 0.003 |  | 1.024 (0.996,1.052) | 0.114 |  | 1.021 (0.994,1.049) | 0.147 | 0.130 | 0.135 | 0.567 |
| Urinary potassium/creatinine ratio | 0.987 (0.976,0.997) | 0.015 |  | 0.985 (0.948,1.022) | 0.435 |  | 0.991 (0.978,1.005) | 0.203 |  | 0.987 (0.958,1.017) | 0.393 |  | 1.004 (0.984,1.023) | 0.725 | 0.211 | 0.103 | 0.908 |
| Serum calcium | 1.011 (1.008,1.015) | <0.001 |  | 1.017 (1.007,1.026) | 0.001 |  | 1.011 (1.007,1.016) | <0.001 |  | 1.011 (1.001,1.021) | 0.036 |  | 1.012 (1.003,1.020) | 0.007 | 0.037 | 0.025 | 0.270 |
| Serum phosphate | 0.993 (0.977,1.009) | 0.384 |  | 1.004 (0.977,1.033) | 0.761 |  | 1.001 (0.979,1.023) | 0.930 |  | 0.944 (0.882,1.009) | 0.093 |  | 1.005 (0.983,1.028) | 0.679 | <0.001 | 0.321 | <0.001 |
| PTH | 1.019 (0.939,1.106) | 0.655 |  | 1.438 (0.925,2.237) | 0.353 |  | 1.041 (1.020,1.062) | <0.001 |  | 1.046 (1.020,1.074) | 0.074 |  | 1.057 (1.041,1.074) | 0.020 | NA | <0.01 | 0.365 |
| 25OHD | 1.004 (1.000,1.007) | 0.026 |  | 1.003 (0.999,1.007) | 0.114 |  | 1.003 (1.000,1.006) | 0.036 |  | 1.013 (0.999,1.028) | 0.075 |  | 1.004 (1.000,1.007) | 0.034 | 0.215 | 0.103 | 0.816 |
| Vitamin C | 1.007 (0.999,1.015) | 0.068 |  | 1.002 (0.979,1.025) | 0.861 |  | 1.006 (0.999,1.012) | 0.087 |  | 1.006 (0.993,1.019) | 0.377 |  | 1.004 (0.996,1.013) | 0.365 | 0.242 | 0.008 | 0.678 |
| CRP | 1.001 (1.000,1.003) | 0.150 |  | 1.002 (0.999,1.004) | 0.205 |  | 1.002 (1.000,1.005) | 0.045 |  | 1.002 (0.996,1.007) | 0.518 |  | 1.002 (1.000,1.004) | 0.099 | 0.09 | 0.080 | 0.689 |
| eGFRcrea | 1.061 (1.041,1.080) | <0.001 |  | 1.085 (1.040,1.133) | <0.001 |  | 1.053 (1.027,1.080) | <0.001 |  | 1.032 (0.961,1.108) | 0.391 |  | 1.065 (1.024,1.107) | 0.002 | <0.001 | <0.001 | 0.249 |
| eGFRcys | 1.035 (1.010,1.061) | 0.010 |  | 1.080 (0.994,1.175) | 0.073 |  | 1.037 (1.008,1.066) | 0.008 |  | 0.984 (0.910,1.064) | 0.688 |  | 1.041 (0.989,1.097) | 0.129 | <0.001 | <0.001 | 0.294 |
| BUN | 0.982 (0.956,1.008) | 0.180 |  | 0.940 (0.883,1.000) | 0.055 |  | 0.963 (0.937,0.990) | 0.007 |  | 1.015 (0.949,1.086) | 0.666 |  | 0.962 (0.932,0.992) | 0.016 | <0.001 | <0.001 | 0.133 |
| Urate | 0.997 (0.994,1.000) | 0.064 |  | 0.996 (0.990,1.002) | 0.212 |  | 0.997 (0.992,1.002) | 0.220 |  | 0.999 (0.987,1.010) | 0.804 |  | 0.999 (0.993,1.005) | 0.812 | <0.001 | <0.001 | 0.725 |
| Testosterone | 0.997 (0.993,1.002) | 0.259 |  | 0.992 (0.985,1.000) | 0.051 |  | 0.994 (0.987,1.001) | 0.072 |  | 0.994 (0.979,1.009) | 0.432 |  | 0.995 (0.988,1.002) | 0.137 | <0.001 | 0.052 | 0.111 |
| Estradiol | 0.999 (0.974,1.024) | 0.915 |  | 1.004 (0.929,1.084) | 0.927 |  | 1.002 (0.974,1.030) | 0.917 |  | 0.986 (0.944,1.030) | 0.541 |  | 1.005 (0.975,1.036) | 0.755 | 0.311 | 0.195 | 0.894 |
| HDL cholesterol | 1.001 (0.999,1.003) | 0.279 |  | 1.001 (0.999,1.004) | 0.338 |  | 1.001 (0.999,1.003) | 0.416 |  | 0.998 (0.993,1.004) | 0.558 |  | 1.001 (0.998,1.003) | 0.502 | 0.023 | 0.017 | 0.699 |
| LDL cholesterol | 1.000 (0.998,1.001) | 0.818 |  | 0.999 (0.997,1.002) | 0.588 |  | 1.000 (0.998,1.002) | 0.854 |  | 0.998 (0.994,1.003) | 0.453 |  | 1.000 (0.998,1.002) | 0.820 | 0.005 | 0.017 | 0.593 |
| Total cholesterol | 1.000 (0.998,1.002) | 0.976 |  | 1.000 (0.997,1.002) | 0.878 |  | 0.998 (0.996,1.001) | 0.178 |  | 1.001 (0.997,1.005) | 0.699 |  | 0.999 (0.997,1.001) | 0.438 | 0.019 | 0.002 | 0.824 |
| Triglycerides | 0.999 (0.997,1.001) | 0.413 |  | 0.997 (0.993,1.001) | 0.188 |  | 1.000 (0.997,1.003) | 0.984 |  | 0.995 (0.990,1.001) | 0.125 |  | 1.000 (0.997,1.003) | 0.867 | 0.029 | 0.023 | 0.297 |
| **Metabolic comorbidities** | | | | | | | | | | | | | | | | | |
| BMI | 1.005 (1.003,1.007) | <0.001 |  | 1.005 (1.000,1.010) | 0.074 |  | 1.004 (1.003,1.007) | 0.006 |  | 1.001 (0.990,1.011) | 0.911 |  | 1.002 (0.996,1.008) | 0.528 | 0.004 | 0.004 | 0.968 |
| Waist circumference | 1.006 (1.002,1.010) | 0.003 |  | 0.999 (0.985,1.014) | 0.915 |  | 1.003 (0.998,1.009) | 0.256 |  | 0.999 (0.987,1.012) | 0.871 |  | 0.999 (0.989,1.008) | 0.776 | 0.035 | 0.106 | 0.324 |
| T2DM | 1.001 (1.000,1.002) | 0.009 |  | 1.000 (0.998,1.002) | 0.864 |  | 1.000 (0.999,1.002) | 0.566 |  | 1.003 (1.000,1.007) | 0.071 |  | 1.000 (0.999,1.002) | 0.715 | 0.005 | 0.005 | 0.226 |
| Fasting glucose | 1.004 (0.999,1.009) | 0.147 |  | 1.009 (0.999,1.018) | 0.072 |  | 1.006 (0.999,1.013) | 0.093 |  | 1.010 (0.996,1.024) | 0.168 |  | 1.006 (1.000,1.012) | 0.065 | 0.154 | 0.071 | 0.219 |
| Fasting insulin | 1.013 (1.005,1.021) | 0.001 |  | 1.024 (1.000,1.049) | 0.061 |  | 1.010 (0.999,1.021) | 0.076 |  | 1.001 (0.975,1.029) | 0.918 |  | 1.029 (1.006,1.054) | 0.022 | 0.398 | 0.395 | 0.348 |
| Glycated hemoglobin | 1.000 (1.000,1.001) | 0.007 |  | 1.000 (0.999,1.001) | 0.602 |  | 1.000 (1.000,1.001) | 0.089 |  | 1.001 (1.000,1.002) | 0.196 |  | 1.000 (1.000,1.001) | 0.290 | <0.001 | 0.060 | 0.047 |
| Hypertension | 1.019 (1.001,1.036) | 0.036 |  | 1.065 (1.018,1.113) | 0.009 |  | 1.025 (1.003,1.047) | 0.026 |  | 1.004 (0.945,1.067) | 0.891 |  | 1.040 (1.001,1.081) | 0.050 | 0.004 | 0.005 | 0.042 |
| DBP | 1.000 (1.000,1.000) | 0.276 |  | 1.000 (0.999,1.001) | 0.892 |  | 1.000 (0.999,1.000) | 0.065 |  | 0.999 (0.998,1.000) | 0.177 |  | 0.999 (0.999,1.000) | 0.050 | <0.001 | <0.001 | 0.530 |
| SBP | 1.000 (1.000,1.000) | 0.548 |  | 1.000 (1.000,1.000) | 0.554 |  | 1.000 (1.000,1.000) | 0.136 |  | 1.000 (0.999,1.000) | 0.670 |  | 1.000 (0.999,1.000) | 0.130 | <0.001 | <0.001 | 0.694 |
| CAD | 1.000 (0.999,1.002) | 0.650 |  | 1.002 (1.000,1.005) | 0.072 |  | 1.002 (1.000,1.003) | 0.057 |  | 1.001 (0.997,1.005) | 0.682 |  | 1.002 (1.000,1.004) | 0.138 | <0.001 | <0.001 | 0.071 |
| Ischemic stroke | 1.003 (0.993,1.012) | 0.613 |  | 1.071 (1.014,1.130) | 0.244 |  | 1.004 (0.997,1.012) | 0.251 |  | 1.007 (0.996,1.017) | 0.357 |  | 1.006 (0.996,1.016) | 0.348 | NA | 0.143 | 0.319 |

**IVW**, inverse variance weighted; **PTH**, parathyroid hormone; **25OHD**, 25-hydroxyvitamin D; **CRP**, C-reactive protein; **GFR**, glomerular filtration rate; **eGFRcrea**, GFR estimated by creatinine; **eGFRcys**, GFR estimated by serum cystatin C; **BUN**, blood urea nitrogen; **BMI**, body mass index; **T2DM**, Type 2 Diabetes Mellitus; **DBP**, diastolic blood pressure; **SBP**, systolic blood pressure; **CAD**, coronary artery disease.
