# Supplementary material for: Lifestyle factors, serum parameters, metabolic comorbidities, and the risk of kidney stones: a Mendelian randomization study
Source: Front Endocrinol (Lausanne). 2023 Sep 22;14:1240171. doi: 10.3389/fendo.2023.1240171 (PMC10560039; doi:10.3389/fendo.2023.1240171)
Supplement: Supplementary file 11 [file Table_5.docx]

Table S5: MR estimating the causal associations between education and the selected mediators.

| Mediator | Method | No of SNPs | β (95% CI) | p value |
| --- | --- | --- | --- | --- |
| BMI | IVW | 203 | -0.252 (-0.327, -0.177) | 5.80e-11 |
|  | MR Egger | 203 | -0.144 (-0.462, 0.174) | 3.75e-01 |
|  | Weighted median | 203 | -0.168 (-0.217, -0.119) | 1.44e-11 |
|  | Simple mode | 203 | -0.198 (-0.353, -0.043) | 1.24e-02 |
|  | Weighted mode | 203 | -0.175 (-0.318, -0.032) | 1.79e-02 |
| Waist circumference | IVW | 213 | -0.122 (-0.167, -0.077) | 7.95e-08 |
|  | MR Egger | 213 | -0.231 (-0.417, -0.045) | 1.59e-02 |
|  | Weighted median | 213 | -0.119 (-0.162, -0.076) | 4.00e-08 |
|  | Simple mode | 213 | 0.024 (-0.048, 0.123) | 7.46e-01 |
|  | Weighted mode | 213 | -0.056 (-0.174, 0.062) | 3.56e-01 |
| Smoking initiation | IVW | 425 | -0.443 (-0.501, -0.385) | 5.11e-51 |
|  | MR Egger | 425 | -0.336 (-0.542, -0.130) | 1.46e-03 |
|  | Weighted median | 425 | -0.382 (-0.441, -0.323) | 4.81e-37 |
|  | Simple mode | 425 | -0.423 (-0.656, -0.190) | 4.41e-04 |
|  | Weighted mode | 425 | -0.374 (-1.474, -0.194) | 6.25e-05 |
| Watching TV (sedentary behavior) | IVW | 430 | -0.603 (-0.636, -0.570) | 2.35e-284 |
|  | MR Egger | 430 | -0.643 (-0.760, -0.486) | 3.45e-24 |
|  | Weighted median | 430 | -0.558 (-0.595, -0.521) | 1.36e-189 |
|  | Simple mode | 430 | -0.708 (-0.882, -0.534) | 1.95e-14 |
|  | Weighted mode | 430 | -0.444 (-0.636, -0.252) | 7.36e-06 |
| T2DM | IVW | 412 | -0.659 (-0.771, -0.547) | 2.17e-31 |
|  | MR Egger | 412 | -0.610 (-1.024, -0.196) | 4.03e-03 |
|  | Weighted median | 412 | -0.574 (-0.692, -0.456) | 9.82e-22 |
|  | Simple mode | 412 | -0.632 (-1.049, -0.215) | 3.16e-03 |
|  | Weighted mode | 412 | -0.588 (-0.960, -0.216) | 2.05e-03 |
