# Supplementary material for: Lifestyle factors, serum parameters, metabolic comorbidities, and the risk of kidney stones: a Mendelian randomization study
Source: Front Endocrinol (Lausanne). 2023 Sep 22;14:1240171. doi: 10.3389/fendo.2023.1240171 (PMC10560039; doi:10.3389/fendo.2023.1240171)
Supplement: Supplementary file 12 [file Table_6.docx]

Table S6: MR estimating the causal associations of each mediator with kidney stones with adjustment for education.

| Mediator | Method | No of SNPs | β (95% CI) | p value |
| --- | --- | --- | --- | --- |
| BMI | IVW | 616 | 0.005 (0.003, 0.007) | 2.18e-07 |
|  | MR Egger | 616 | 0.003 (-0.002, 0.008) | 0.166 |
|  | Weighted median | 616 | 0.005 (0.002, 0.008) | 1.42e-03 |
|  | Simple mode | 616 | 0.008 (-0.001, 0.017) | 0.109 |
|  | Weighted mode | 616 | 0.003 (0.001, 0.005) | 0.366 |
| Waist circumference | IVW | 458 | 0.004 (0.002, 0.006) | 6.42e-05 |
|  | MR Egger | 458 | 0.004 (-0.001, 0.009) | 0.132 |
|  | Weighted median | 458 | 0.003 (-0.0003, 0.006) | 0.059 |
|  | Simple mode | 458 | 0.002 (-0.007, 0.011) | 0.746 |
|  | Weighted mode | 458 | 0.003 (-0.003, 0.009) | 0.352 |
| Smoking initiation | IVW | 91 | 0.004 (0.001, 0.007) | 0.028 |
|  | MR Egger | 91 | 0.008 (-0.008, 0.024) | 0.324 |
|  | Weighted median | 91 | 0.001 (-0.003, 0.005) | 0.587 |
|  | Simple mode | 91 | 0.001 (-0.011, 0.013) | 0.896 |
|  | Weighted mode | 91 | 0.002 (-0.011, 0.015) | 0.802 |
| Watching TV (sedentary behavior) | IVW | 170 | 0.007 (0.003, 0.010) | 2.84e-04 |
|  | MR Egger | 170 | 0.005 (-0.011, 0.021) | 0.545 |
|  | Weighted median | 170 | 0.006 (-0.001, 0.001) | 0.026 |
|  | Simple mode | 170 | 0.003 (-0.012, 0.018) | 0.734 |
|  | Weighted mode | 170 | 0.003 (-0.010, 0.018) | 0.695 |
| T2DM | IVW | 128 | 0.001 (0.0002, 0.002) | 0.016 |
|  | MR Egger | 128 | 0.001 (-0.002, -0.003) | 0.642 |
|  | Weighted median | 128 | 0.0003 (-0.001, 0.002) | 0.662 |
|  | Simple mode | 128 | 0.005 (0.001, 0.009) | 0.028 |
|  | Weighted mode | 128 | 0.0001 (-0.002, 0.017) | 0.914 |
